# Etomidate Vaping: An Emerging Substance Use Pattern in Singapore - A Case Series

**DOI:** 10.1192/j.eurpsy.2026.10865

**Published:** 2026-07-31

**Authors:** C. Q. Low, A. Yiyang, K. Gomathinayagam

**Affiliations:** Institute of Mental Health, Singapore, Singapore

## Abstract

**Introduction:**

The emergence of etomidate vaping represents a novel form of substance misuse, particularly concerning in Singapore’s strict regulatory environment. Etomidate is a short-acting intravenous hypnotic agent used in anaesthesia, but recent reports from Hong Kong and Mainland China describe recreational misuse via vaping devices. Singapore has reported over 70 cases of etomidate-laced e-vaporisers investigated or prosecuted locally, with several associated fatalities.

**Objectives:**

To describe the first documented cases of etomidate vaping in Singapore, examining clinical presentations, treatment approaches, and outcomes to raise awareness among healthcare providers and inform public health responses.

**Methods:**

We present two cases of etomidate vaping encountered at the Institute of Mental Health, Singapore’s tertiary psychiatric hospital, between October 2024 and August 2025. Cases were selected based on self-reported use of etomidate-containing vaping products and availability of comprehensive clinical documentation. Clinical information was extracted from electronic medical records, including demographic details, substance use patterns, clinical presentations, treatment interventions, and follow-up outcomes.

**Results:**

Two cases were identified: one male (age 33) and one female (age 26). Both presented with distinctive clinical features including trance-like states and behavioural disturbances. The male patient showed sustained remission following structured inpatient treatment, while the female patient initially maintained abstinence but later experienced relapse precipitated by occupational stress. Notably, withdrawal symptoms were absent during initial cessation but emerged with repeated use patterns in the female patient, suggesting evolving dependence potential. Both cases demonstrated rapid functional impairment and significant financial expenditure within short timeframes.

**Conclusions:**

Etomidate vaping represents an emerging public health threat requiring immediate healthcare system adaptation, including enhanced screening protocols, specialised laboratory testing capabilities, and modified treatment approaches. The contrasting outcomes between inpatient and outpatient management highlight the need for intensive engagement strategies, whilst the evolution of withdrawal symptoms with repeated use suggests increasing dependence potential over time.

**Disclosure of Interest:**

None Declared

